# THE CZECH FUGL–MEYER ASSESSMENT FOR POST-STROKE SENSORIMOTOR FUNCTION: TRANSLATION AND CROSS-CULTURAL ADAPTATION AND VALIDATION

**DOI:** 10.2340/jrm.v57.43010

**Published:** 2025-05-07

**Authors:** Barbora KOLÁŘOVÁ, Petra GAUL-ALÁČOVÁ, Nicole MUSILOVÁ, Anna MAJEROVÁ, Margit ALT MURPHY

**Affiliations:** 1 Department of Clinical Rehabilitation, Faculty of Health Sciences, Palacký University, Olomouc, Czech Republic; 2 Department of Rehabilitation, University Hospital, Olomouc, Czech Republic; 3 Department of Neurology, Faculty of Medicine and Dentistry, Palacký University Olomouc, Czech Republic; 4 Department of Clinical Neuroscience, Institute of Neuroscience and Physiology, Sahlgrenska Academy, University of Gothenburg, Gothenburg, Sweden; 5 Department of Rehabilitation and Health, Institute of Neuroscience and Physiology, Sahlgrenska Academy, University of Gothenburg, Gothenburg, Sweden

**Keywords:** cross-cultural comparison, lower extremity, stroke, sensorimotor assessment, translation, upper extremity, validation

## Abstract

**Objective:**

To ensure wider use of the internationally recommended Fugl–Meyer Assessment (FMA) of sensorimotor function for people with stroke, official translations of the scale are needed. This study aimed to perform a translation and cross-cultural adaptation/validation of the FMA into the Czech language.

**Design:**

Translation and cross-cultural adaptation/validation.

**Subjects/Patients:**

Five clinical experts and 1 external expert participated as reviewers; 11 individuals with stroke in the early subacute phase were included in the pilot testing.

**Methods:**

A standardized process using forward–backward translations, expert panel reviews, and pilot testing between and within the raters (inter- and intra-rater reliability) were employed to ensure conceptual, semantic, and operational validity of the new Czech FMA. Agreement between raters was assessed in 11 individuals with stroke on 2 consecutive days at University Hospital Olomouc by using Svensson’s rank-based statistics.

**Results:**

Percentage of agreement between and within raters ranged between 70–100% and 55–100%, respectively. Systematic disagreements, found in 7 out of 96 FMA items, were discussed and revised in the final version.

**Conclusion:**

The Czech FMA offers a more unified and standardized assessment of sensorimotor impairment in clinical and research settings. This will improve stroke rehabilitation care and allow for wider international collaboration.

## LAY ABSTRACT

The Fugl–Meyer Assessment is the number one scale recommended for assessment of motor and sensory function in people with stroke. For correct clinical use official validated translations are needed. This study performed a translation and cross-cultural adaptation of the Fugl–Meyer Assessment into Czech. A standardized protocol with forward–backward translation, expert consensus, and pilot testing in 11 individuals with stroke were used to ensure conceptual, semantic, and operational consistency. The results of pilot testing showed good agreement and allowed final adjustments in 7 problematic test items. The cross-culturally adapted Czech Fugl–Meyer Assessment is now available and provides a more unified and standardized assessment of sensorimotor impairment after stroke in both clinical and research settings.

Stroke ranks as 1 of the top 3 causes of non-traumatic death and long-term disability worldwide (1, 2). The Czech Republic is among the countries with the highest incidence of stroke in the world (3). In 2021, approximately 291 patients per 100,000 population were hospitalized for stroke (4). Despite the reduction in the overall incidence and mortality in the Czech Republic, which follows the global trend of this disease (3, 5), the ageing population results in an increasing number of stroke survivors living with some kind of residual deficit.

One of the most common clinical manifestations of stroke is hemiparesis, which particularly limits voluntary control of upper and lower limb movement contralateral to the lesion (6). This leads to impaired postural control, gait, or upper extremity function, limiting the patient’s independence in daily activities (2, 6). The central goal of rehabilitation is to maximize independence in activities of daily living and to improve the quality of life. To evaluate these goals, it is necessary to use reliable and validated tools for assessment of stroke severity and impairment.

Worldwide, one of the most widely used clinical tools for the assessment of sensorimotor function after stroke is the Fugl–Meyer Assessment (FMA) (7–9). The FMA, recognized as the gold standard in clinical and research settings, demonstrates high validity and reliability in the subacute (10, 11) and chronic stages of stroke (6, 12, 13).

The FMA assesses motor functions at the impairment level in respect of the International Classification of Functioning, Disability and Health (ICF) (14). The international consensus recommends including the FMA in all clinical trials in people with stroke (15) and as a key assessment to evaluate the effectiveness of rehabilitation (12). The FMA has been shown to be sensitive in capturing changes after therapeutic interventions, such as robotic rehabilitation (16), virtual reality (17), mental training (18), constrained induced movement therapy (19), and pharmacological treatment (20).

The FMA is an observational ordinal rating scale for evaluating upper (FMA-UE) and lower extremity (FMA-LE) sensorimotor functions (21). The scoring of each test item is clear and standardized, no specific tools are needed, and the administration time is relatively short, which makes the scale easily accessible and clinically feasible (22). This scale is commonly used by physiotherapists and other professionals with appropriate training (medical doctors, occupational therapists). An official version of the FMA in the Czech language is needed to ensure access to nationwide standardized assessment procedures, evaluate outcomes of stroke care within the Czech Republic, and promote international collaboration. It is important to follow standardized translation and cross-cultural adaptation procedures to ensure that the translated version of the scale agrees with the established construct validity and reliability of the original scale and will allow comparisons over time, across countries, and across organizations (23). The official translations of the FMA are available for non-profit clinical or research use in several languages (https://www.gu.se/en/neuroscience-physiology/fugl-meyer-assessment) (10, 11, 24–29).

The aim of this study was to translate and create an official cross-culturally adapted Czech FMA for the assessment of upper and lower extremity sensorimotor function in people with stroke.

## METHODS

Translation and cross-cultural adaptation encompass a process of both language and cultural adaptation to maintain the content validity of the assessment at a conceptual level across different cultures, languages, and countries (30, 31). The translation and cross-cultural adaptation process used within this study was based on the guidelines recommended by Beaton et al. (30) and more recently by Sousa and Rojjanasrirat (31). A standardised forward and backward translation combined with expert reviews was performed in accordance with Barbosa et al. (28) or Busk et al. (24), who further specified slight nuances that are more suitable for the FMA, as this assessment is not administered by the patients themselves, but by trained professionals. The whole process for cross-cultural adaptation was initiated after approval from the curator of the original FMA at the University of Gothenburg. An interdisciplinary team from the Stroke Research Group of Palacký University and University Hospital Olomouc conducted the translation, revision, and cross-cultural validation of pre-final version by pilot testing in post-stroke patients to identify possible disagreements in the pre-final version. The University of Gothenburg provided regular consultations during the entire process.

### Translation process

**Fig. 1:**
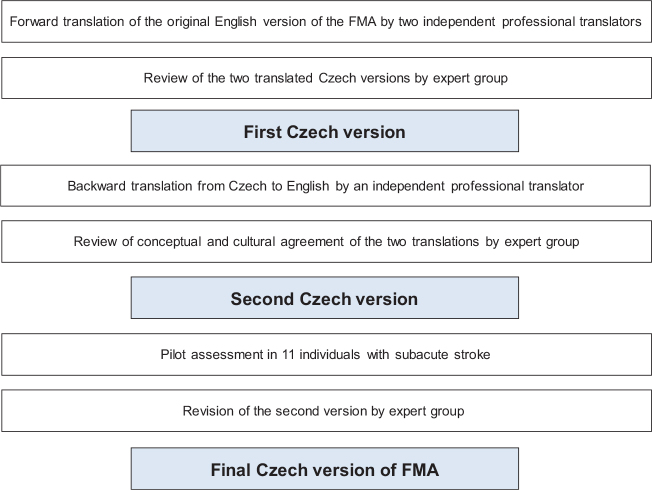
Flowchart of translation and cross-cultural adaptation process.

The original version of the FMA (available at https://www.gu.se/en/neuroscience-physiology/fugl-meyer-assessment) was used for the translation (21). A standardized forward and backward translation combined with expert reviews was performed to ensure conceptual, semantic, and operational equivalence (28, 30, 31). The entire process is shown in Fig. 1. Two professional translators (native speakers of the target language), fluent in both English and Czech, performed the forward translation independently. These translations were compared, to draft the first Czech version, by the expert group consisting of another professional translator, 3 physiotherapists, and 1 medical doctor, all specialized in neurorehabilitation. The first version was translated back to English by another professional bilingual translator (native in English and fluent in Czech) and reviewed by the University of Gothenburg expert (MAM) and 2 Czech clinical researchers (BK and PGA), fluent in English and with knowledge of FMA, to ensure semantic, idiomatic, and conceptual equivalence with the original version. The 2 versions were re-evaluated and the second Czech version of FMA was drafted.

### Pilot testing of the pre-final version

Pilot testing of the pre-final 2nd version was conducted to identify potential disagreements within and between raters when the FMA was used in the Czech context. In this step, 11 participants with stroke were requited from the Rehabilitation Department of University Hospital Olomouc, which is part of the Comprehensive Stroke Centre at University Hospital Olomouc. The inclusion criteria were: first ever stroke verified by CT/MRI scan, lower or/and upper limb paresis due to stroke (as evaluated and documented by a medical doctor specialized in Rehabilitation and Physical Medicine), age 18–80 years, sufficient cognitive function to understand the physiotherapist’s instructions during the FMA assessment, able to provide informed consent and to understand the purpose of the study. Exclusion criteria were: recurrent stroke, other neurological diseases, cognitive, visual, or/and hearing deficit, or aphasia preventing cooperation during the assessment of the FMA. The screening for eligibility was performed by the medical doctor within the first 2 weeks after stroke onset in the Rehabilitation Department. All patients admitted during this period were screened for potentially meeting the inclusion criteria. The research was performed in accordance with the principles stated in the Declaration of Helsinki and was approved by the Ethics Committee of the Faculty of Health Sciences, Palacký University in Olomouc (UPOL-1703/1040-2020). All participants who expressed their agreement to participate in the study signed an informed consent.

The full FMA for the upper and lower extremities, including the non-motor domains of sensation, range of motion, and pain, was carried out by 2 experienced physiotherapists, on 2 consecutive days. Both assessors read properly the Czech version of the FMA and watched the instructional videos with English subtitles (https://www.gu.se/en/neuroscience-physiology/fugl-meyer-assessment). Any unclarities were discussed with a certified English interpreter and a native speaker to avoid any potential impact of misunderstanding or misinterpretation on the study results. In addition, both assessors underwent mutual training sessions on themselves and assessed 3 patients (not included in this study) prior to the study. On the first day, 1 physiotherapist was the lead assessor, who instructed the patient, while the other physiotherapist observed the patient’s performance. Both therapists scored the patient’s performance on each tested item independently. The next day, the assessors’ roles were switched. The assessors did not communicate with each other regarding the assessment during or after the evaluation; however, the observing assessor could ask the patient to repeat a movement whenever necessary for scoring (if they had not had enough time to complete it or could not see the performance properly). Scoring in each FMA domain was based on direct observation of performance. The entire assessment lasted approximately 30 min. The assessment protocols were stored in sealed envelopes immediately after the assessment and were only opened after all participants had been assessed (24, 28).

Elisabeth Svensson’s rank-based statistical method was used. This statistical method is particularly designed for ordinal data to evaluate the inter-rater and intra-rater reliability (32, 33). This method quantifies the type and the extent of observed systematic and non-systematic inter- and intra-rater disagreements (33).

The percentage of agreement (PA%) was calculated. An agreement of ≥ 70% was considered satisfactory and ≥ 90% excellent (24). Systematic disagreement between assessors was evaluated by relative position (RP), which determines whether scores are distributed systematically towards lower or higher values, and by relative concentration (RC), which determines whether scoring is concentrated towards the central category compared with the other categories. RP and RC take values from –1 to 1, where 0 means that there was no difference between the assessors. A value equal or greater than 0.1 is considered as a potential disagreement. Relative rank variation (RRV) demonstrates non-systematic random disagreement caused by individual variability and values < 0.1 indicate that the random error is negligible. The results of the analyses were used to identify which items and in which direction were scored differently by the 2 assessors. These items were discussed and agreed on in the expert group and with the external expert to improve coherence and interpretability.

## RESULTS

### Forward and backward translation

Minor linguistic differences were found between the two forward translations, mostly regarding nomenclature of anatomical terms or positions, similar to the results in Cecchi et al. (25) and Barbosa et al. (28). After a few contextual modifications considering the clinical terminology used in the Czech Republic by physical and occupational therapists as well as medical doctors, consensus was reached for the 1st Czech version of FMA. In the next stage, the 1st version was compared with the back-translated version. Only minor semantic discrepancies, which did not have any effect on the meaning, were found and revised for the finalization of the 2nd Czech version of the FMA.

### Pilot clinical testing

Of 27 patients who were screened for participation in the cross-cultural adaptation of the Czech FMA, 13 were eligible for participation in this study. However, 1 was subsequently excluded due to early discharge from hospital before the second day of assessment, and 1 was excluded due to clinical complications during hospitalization. Pilot testing and final analysis was then based on data collected from 11 participants in an early subacute stage (Table I).

**Table I:** Demographic and clinical characteristics of the participants, *n* = 11

Age (years), median (min/max)	69 (42/79)
Male, *n* (%)	6/5 (54.5%)
Stroke type and location, *n*	
Ischaemic middle cerebral artery	9
Ischaemic anterior cerebral artery	1
Ischaemic thalamus	1
Lesion in the right/left hemisphere, *n*	3/8
Days after stroke, median (min/max)	24 (17/33)
Modified Rankin scale, median (min/max)	4 (3/4)
Functional Ambulation Category, median (min/max)	3 (1/5)
Barthel Index, median (min/max)	65 (50/95)
Fugl–Meyer Assessment: UE (0–66), median (min/max)	41 (11/62)
Fugl–Meyer Assessment: LE (0–34), median (min/max)	21 (19/25)

UE: upper extremity; LE: lower extremity.

The percentage of agreement between assessor A and assessor B (inter-rater agreement) ranged from 73% to 100% for each item of the FMA-UE and FMA-LE, which is classified as a sufficient level of agreement (Tables SI and SII). No systematic or random disagreements were found between assessors (inter-rater agreement).

A minor disagreement was found within assessors between day 1 and day 2 (intra-rater agreement). For FMA-UE, the intra-rater agreement varied between 55% and 100% and was observed to be < 70% in 9 motor items and 1 non-motor item (Table SI). Statistically significant systematic disagreement (CI did not include a zero value and the RP or RC was ≥ 0.1) was present in 3 items: *A.III: Movement mixing synergies (shoulder flexion and pronation–supination)*, and for *I: Passive joint motion (shoulder external rotation).* For FMA-LE, the intra-rater agreement was in the range of 45–100%, where agreement < 70% was present in 3 motor items and 2 non-motor items (Table SII). Statistically significant systematic disagreement was found for *A*.*II: Voluntary movement in synergies (ankle dorsal flexion)* and in *A.III: Movement in mixed synergies (ankle dorsiflexion)*, *H: Sensation (position toe)*, and *I: Passive joint motion* (*foot pronation)*. No random disagreements were detected.

### Final Czech version

The identified disagreements from the pilot testing were reviewed and discussed with the assessors in the expert group. The main final adjustments agreed in the expert groups are described below.

For the item *III: Movement mixing synergies (pronation–supination*), the translation of expression “no pronation/supination, starting position impossible” was revised and changed from “bez pronace/supinace, výchozí pozice nemožná” (meaning without pronation/supination) to “žádná pronace/supinace, výchozí pozice nemožná” (with a more exact meaning of any or no pronation/supination). The translation of the expression “elbow flexion” was revised and changed from “flexe lokte” to “flexe v lokti” for better understanding.

Furthermore, according to the assessors’ suggestions, the expression “Volitional movement mixing synergies” was changed from “Volní pohyby smíšené synergie” to “Volní pohyby kombinující synergie” (volitional movement combining synergies) in both FMA-UE and FMA-LE. On the FME-UE, the expression ‘Volitional movement within synergies, without gravitational help” was also changed from “…bez pomoci gravitace” (without the help of gravity) to “…bez pomoci proti gravitaci” (without help against gravity) to align with the terms commonly used in Czech, for example, in manual muscle strength testing. The expression “no support to wrist” was changed from “žádná opora o zápěstí” to “opora zápěstí není poskytnuta” (support at wrist is not provided), to align with the expression used in the lower extremity section. The expression for Coordination/speed subscale in FMA-UE “after one trial with both arms” was changed from “nejdříve jeden zkušební test oběma rukama” to “zkušební test jednou a následně druhou rukou”, as in Czech “oběma rukama” means precisely *simultaneously performed with both arms*, but the test trial before scoring needed to be performed first with the non-paretic and then with the paretic upper limb.

These final modifications were sent to the researchers at the University of Gothenburg in May 2022, who reviewed and approved these proposed adjustments. The last revision of the FMA protocol and application manual resulted in the final Czech version of the FMA, which is now available on the website (https://www.gu.se/en/neuroscience-physiology/fugl-meyer-assessment).

## DISCUSSION

This study provides an official translation and cross-cultural adaptation of the original FMA assessment protocol from English into Czech. The preliminary inter- and intra-rater reliability evaluation showed sufficient inter-rater reliability for both FMA-UE and FMA-LE. The official protocol of the Czech FMA will ensure standardized assessment of sensorimotor impairment in patients at all stages after stroke.

The FMA is the most frequently used measure in stroke rehabilitation trials (7–9, 34) and the motor domains of the FMA have shown high validity, reliability, and responsiveness for stroke populations (35). It has been used in both subacute (10, 11) and chronic patients after stroke (6, 12, 13, 36). Ceiling effect has been observed in patients with mild motor deficits (37, 38), while patients with severe motor deficits may demonstrate a floor effect (36, 39). The FMA is considered a valid tool for assessment of motor impairment severity poststroke in clinical practice and rehabilitation research (40). Given the strong evidence of measurement properties, the FMA is included in the core set of European evidence-based recommendations for Clinical Assessment of the Upper Limb in Neurorehabilitation (52) and in the international consensus-based core recommendations of the Stroke Recovery and Rehabilitation Roundtable (53).

In the current study, both motor and non-motor subscales of FMA were translated and further analysed for item-level reliability to identify any problematic items where disagreements were found between the assessors. The translation of both motor and non-motor subscales will provide access to the complete protocol of Czech FMA in clinical and research settings.

The FMA assesses impairment according to ICF, and together with other assessments of activity level will provide deeper insight into the course of motor recovery after stroke, which can facilitate a more precise rehabilitation strategy (41). From both a clinical and scientific perspective, the Czech FMA will provide an opportunity to compare the results between clinical sites nationally to improve the quality of care and in the long term also internationally when included in European or global databases compiling larger datasets from multiple sites and countries to better describe stroke severity and recovery patterns in all post-stroke stages (28, 42). Additionally, the use of the FMA allows clinicians and researchers to determine whether the change in the FMA scores, due to intervention, represents clinically significant improvement, as minimal clinically significant differences are well established for both FMA-UE and FMA-LE (43–45). The FMA also has potential to predict motor recovery early post-stroke (46). The FMA-UE has been used to predict upper limb motor recovery within 72 h after stroke onset (47–49).

The pilot testing showed excellent to sufficient inter-rater reliability, as the level of agreement was for both FMA-UE and FMA-LE on all items in the range of 70–100%. These results agreed with previously reported intra- and inter-rater agreements of the Spanish, Danish, and Italian translated FMA versions (70–100%) (10, 11, 24, 50). Excellent inter-rater reliability with high ICC values was demonstrated for the German version of the FMA, although the percentage of inter-rater agreement at item level was reported to be lower (median 77%, range 44–100%) (51). Even the Korean (26), Romanian (27), or Urdu (29) versions of the FMA have been validated for clinical use on the basis of good to excellent reliability and validity.

No statistically significant systematic disagreements were found in any items for the inter-rater reliability in our study. However, the intra-rater reliability (agreement between the assessments done by the same assessor 1 day apart) showed disagreements below 70% on some items. A similar trend with disagreements detected between the first and second assessment for 2 items (ankle dorsiflexion during flexor synergy and the normal reflex activity) was reported in the study of Hernandéz et al. (11). The shifts towards higher scores on the second day may indicate possible spontaneous recovery at an early stage of stroke (11). For future studies conducted on participants in the early subacute stage, the reassessment should preferably be conducted in a shorter time interval, e.g. within the same day, to avoid mixing potential spontaneous improvements with differences in assessment scores.

Both assessors underwent prior training on the FMA by watching the instructional videos and conducting trial assessments. We agree with the statement of Busk et al. (24) that proper practice with testing and scoring prior to assessment itself is essential. We also acknowledge that performing all parts of the FMA might be tiring for the patient in the early subacute phase and here the upper and lower extremity assessments could preferably be performed separately in clinical settings. However, none of the participants in the current study reported complaints of exhaustion.

The cross-cultural adaptation of the Czech FMA conducted in this study was sufficient to identify problematic items to improve the final Czech FMA, although further studies with a larger sample size are necessary to confirm its validity and reliability in the Czech context.

The use of the FMA worldwide has great significance for clinicians, researchers, and educators in the field of stroke rehabilitation. The current study adds a new official Czech translation, which will allow for wider standardized use of the scale. The FMA is easy to use and does not need any special equipment (28), which makes it easy to use in research and clinical practice.

### Strength and limitations

The strength of this current study is in a comprehensive standardized translation and cross-cultural validation procedure carried out in 8 steps. It included 2 forward translations, their comparison, and finalization of the first draft, which was then subjected to backward translation, compared with the original, with step-wise reviewing by a proof-reader as well as a bilingual physiotherapist. The final translation was subsequently tested in a pilot study on 11 post-stroke patients to reveal any possible discrepancies in all the evaluation steps. An important limitation of the current study is that this preliminary reliability evaluation of the Czech FMA provides only limited information on reliability, as only a minor range of possible scores of the FMA were covered in this small sample.

### Conclusions

The official translation of the Czech FMA for both the upper and lower extremities was developed in this study. The official Czech FMA is now available for clinical, educational, and scientific use. Systematic post-stroke assessment of sensorimotor function is essential to gain a better understanding of post-stroke motor recovery and to select an appropriate rehabilitation strategy more precisely. The FMA can be successfully used to classify the stroke severity level and to predict recovery outcome. The Czech FMA will allow for appropriate and unified assessment of motor impairment post-stroke and a comparison of the outcomes across countries worldwide. From this perspective, internationally comparable outcomes from the FMA have the potential to add pieces of evidence to improve the quality of rehabilitation in all stages after stroke.

## Supplementary Material

THE CZECH FUGL–MEYER ASSESSMENT FOR POST-STROKE SENSORIMOTOR FUNCTION: TRANSLATION AND CROSS-CULTURAL ADAPTATION AND VALIDATION
