# Supplementary material for: THE CZECH FUGL–MEYER ASSESSMENT FOR POST-STROKE SENSORIMOTOR FUNCTION: TRANSLATION AND CROSS-CULTURAL ADAPTATION AND VALIDATION
Source: J Rehabil Med. 2025 May 7;57:43010. doi: 10.2340/jrm.v57.43010 (PMC12079045; doi:10.2340/jrm.v57.43010)
Supplement: THE CZECH FUGL–MEYER ASSESSMENT FOR POST-STROKE SENSORIMOTOR FUNCTION: TRANSLATION AND CROSS-CULTURAL ADAPTATION AND VALIDATION [file JRM-57-43010-s1.pdf]

Supplementary material has been published as submitted. It has not been copyedited, or typeset by Journal of Rehabilitation Medicine

Table SI. The inter- and intra-rater agreement for Fugl-Meyer Assessment of Upper Extremity

Test items	Inter-rater agreement						Intra-rater agreement					
	1st day			2nd day			Rater A			Rater B		
	Rater A vs B			Rater A vs B			1st vs 2nd day			1st vs 2nd day		
**A. Upper extremity**	**PA**	**RP**	**RC**	**PA**	**RP**	**RC**	**PA**	**RP**	**RC**	**PA**	**RP**	**RC**
**I. Reflex activity**												
Flexors	100			100			82	0.18		82	0.18	
Extensors	100			100			82	0.18		82	0.18	
**II. Within synergies**												
Retraction	82	0	0	91	-0.09	0	91	0.09	0	82	0	0
Elevation	73	-0.09	0	82	-0.18	0	73	0.09	0	82	0	0
Abduction	91	-0.12	-0.11	73	0.09	0	82	0	0	73	0.21	0.11
External rotation	91	-0.08	0.04	82	-0.17	0.12	**64**	0.17	-0.12	73	0.08	-0.04
Elbow flexion	100			91	-0.09	0	91	0.09	0	100		
Supination	73	-0.06	-0.10	91	-0.07	0.09	**64**	0.12	0.01	**64**	0.12	0.21
Adduction/rot	100			100			100			100		
Elbow extension	100			100			100			100		
Pronation	73	0.07	-0.09	100			73	0.07	-0.09	82	0	0
**III. Mixing synergies**												
Hand to lumbar spine	91	0.08	-0.04	82	0.18	0	**64**	-0.04	0.14	91	0.03	0.12
Shoulder flexion	91	-0.06	-0.10	82	-0.02	0.16	73	0.17	0.14	**64**	0.23	**0.45***
Pronation – supination	82	-0.13	0.18	82	-0.15	0.15	73	0.18	-0.05	**55**	0.20	**-0.06***
**IV. Little/no synergy**												
Shoulder abduction	100			100			**64**	0.12	0.04	**64**	0.12	0.04
Shoulder flexion	91	-0.09	0	91	-0.07	0.1	82	0.13	-0.23	82	0.15	-0.11
Pronation - supination	91	-0.07	0.07	100			**64**	0.14	0.05	73	0.22	-0.02
**B. Wrist**												
Stability 90°	91	0.03	0.12	91	0.03	0.12	100			100		
Repeated flexion	91	0.07	-0.07	73	0.08	-0.04	73	0.05	0.11	91	0.04	0.11
Stability 0°	91	0.07	-0.10	100			91	0.07	-0.10	100		
Repeated flexion	91	0.66	-0.09	82	0	0	73	0.05	0.11	82	-0.02	0.18
Circumduction	91	0.06	0.10	100			73	0.17	0.33	82	0.12	0.22
**C. Hand**												
Mass flexion	**91**	0.03	0.11	100			**64**	0.11	0.06	73	0.08	-0.05
Mass extension	91	0.05	0.11	82	-0.15	0.15	82	0.15	-0.15	91	-0.05	-0.11
Hook grasp	91	0.04	0.11	100			91	0.04	0.11	100		
Thumb adduction	91	0.04	0.11	82	-0.04	0.21	73	0.13	-0.23	73	0.07	-0.07
Pincer grasp, opposition	91	0.02	0.11	91	0.07	-0.08	82	-0.04	0.21	100		
Cylinder grasp	100			91	-0.08	0.05	91	0.08	-0.05	100		
Spherical grasp	100			91	0.08	-0.05	91	-0.08	0.05	100		
**D. Coordination**												
Tremor	82	0	0	100			82	0.03	-0.16	82	0.02	-0.16
Dysmetria	91	-0.08	0.04	100			73	0.08	-0.04	**64**	0.17	-0.09
Time	100			100			73	0.11	0.23	73	0.11	0.23
**H. Sensation**												
Touch arm	100			100			100			100		
Touch hand	100			100			91	0.09	0	91	0.09	0
Position shoulder	100			100			91	0.09		91	0.09	
Position elbow	100			100			73	0.09	0	73	0.09	0
Position wrist	100			100			73	0.11	0.20	73	0.11	0.20
Position thumb	91	0.07	-0.11	100			82	0.02	0.11	91	0.09	0
**I. Joint motion**												
Shoulder flexion	100			100			82	0.00	0	82	0.00	0
Shoulder abduction	91	0.09	0	91	0.09	0	82	0.18	0	82	0.18	0

Shoulder ext/rotation	82	0	0	82	-0.18	0	73	**0.27***	0	73	0.09	0
Shoulder int/rotation	91	-0.09	0	73	-0.21	-0.11	73	0.09	0	**64**	0.21	0.11
Elbow flexion	100			100			91	0.11		91	0.09	
Elbow extension	91	-0.09	0	100			91	0.09		82	0.18	
Pronation	100			100			91	0.09		91	0.09	
Supination	100			100			100			100		
Wrist flexion	82	0	0	100			91	0.09		91	0.09	
Wrist extension	100			100			82	0	0	82	0	0
Fingers flexion	100			100			100			100	0	
Fingers extension	91	-0.09	0	91	-0.09	0	100			100		
**J. Joint Pain**												
Shoulder flexion	91	0.08	-0.06	100			82	0	0	91	-0.08	0.06
Shoulder abduction	100			100			82	0	0	82	0	0
Shoulder ext/rotation	100			91	0.08	-0.05	82	0.17	-0.12	73	-0.08	0.06
Shoulder int/rotation	100			91	-0.09		91	0.09		100		
Elbow flexion	100			100			82	-0.01	-0.10	82	0.01	0.10
Elbow extension	100			100			100			100		
Pronation	100			100			91	0.09		91	0.09	
Supination	100			100			91	0.09		91	0.09	
Wrist flexion	100			100			100			100		
Wrist extension	100			100			91	0.09		91	0.09	
Fingers flexion	100			100			100			100		
Fingers extension	100			100			100			100		

Abbreviation: vs, versus; RP, relative position (systematic disagreement); RC, relative concentration (systematic disagreement); RV, relative variance (random variance); * statistically significant disagreement; disagreements and PA values < 70 are shown in bold.

Table SII. The inter- and intra-rater agreement for Fugl-Meyer Assessment of Lower Extremity.

	Inter-rater agreement						Intra-rater agreement					
	1st day			2nd day			Rater A			Rater B		
	Rater A vs B			Rater A vs B			1st vs 2nd day			1st vs 2nd day		
**E. Lower extremity**	**PA**	**RP**	**RC**	**PA**	**RP**	**RC**	**PA**	**RP**	**RC**	**PA**	**RP**	**RC**
**I. Reflex activity**												
Flexors	100			100			100			100		
Extensors	100			100			91	0.09	0	91	0.09	0
**II. Synergies**												
Hip flexion	100			100			91	0.09		91	0.09	
Knee flexion	100			100			100			100		
Foot dorsal flexion	82	0.18	0	100			73	**0.27***	0	91	0.10	0
Hip extension	100			100			100			100		
Hip adduction	91	0.09		100			91	0.09		100		
Knee extension	91	0.09		100			91	0.09		100		
Foot plantar flexion	100			91	-0.09	0.00	82	0.18	0	73	0.09	0
**III. Mixed synergies**												
Knee flexion	100			100			100			100		
Ankle dorsiflexion	82	0	0	100			73	**0.27***	0	73	**0.27***	0
**IV. Little/no synergy**												
Knee flexion to 90°	100			91	-0.07	-0.11	**55**	0.07	0.11	100		
Ankle dorsiflexion	91	0.07	-0.07	91	0.08	-0.05	82	0.12	0.07	82	0.11	0.06
**F. Coordination**												
Tremor	100			100			**64**	0.02	-0.13	**64**	0.02	-0.13
Dysmetria	100			91	-0.06	-0.11	73	0.08	-0.04	**64**	0.02	-0.15
Time	100			100			**55**	0.17	0.18	**55**	0.17	0.18
**H. Sensation**												
Light touch leg	100			100			73	0.09	0	73	0.09	0
Light touch foot	100			100			82	0	0	82	0	0
Position hip	91	-0.09		100			100			91	0.09	
Position knee	100			100			100			100		
Position ankle	100			100			100			100		
Position toe	82	0.06	-0.96	100			73	**0.27***		82	0.18	
**I. Joint motion**												
Hip flexion	100			91	-0.09		100			91	0.09	
Hip abduction	100			100			91	0.09		91	0.09	
Hip external rotation	91	0.09	0	100			82	0	0	91	0.09	0
Hip internal rotation	82	-0.06	0.15	82	0.12	0.11	**64**	0.17	-0.10	82	0	0
Knee flexion	100			91	-0.09		100			91	0.09	
Knee extension	100			100			100			100		
Ankle dorsiflexion	82	-0.12	-0.11	91	0.08	-0.04	73	0.21	0.11	82	0	0
Ankle plantar flexion	91	0.09	0	100			82	0.18		91	0.09	
Foot pronation	82	-0.18	0	100			**45**	**0.18***	0	**45**	**0.36***	0
Foot supination	91	0.09		100			91	0.09		100		
**J. Joint pain**												
Hip flexion	91	-0.09		100			91	0.09		100		
Hip abduction	100			100			91	0.09		91	0.09	
Hip external rotation	100			100			100			100		
Hip internal rotation	100			100			100			100		
Knee flexion	100			100			100			100		
Knee extension	100			100			91	0.09		91	0.09	
Ankle dorsiflexion	100			100			100			100		
Ankle plantar flexion	100			100			100			100		
Foot pronation	100			100			100			100		
Foot supination	100			100			100			100		

Abbreviation: vs, versus; RP, relative position (systematic disagreement); RC, relative concentration (systematic disagreement); RV, relative variance (random variance); * statistically significant disagreement; disagreements and PA values < 70 are shown in bold.
